# Insulin-Like Growth Factor-1 Differentially Modulates Glutamate-Induced Toxicity and Stress in Cells of the Neurogliovascular Unit

**DOI:** 10.3389/fnagi.2021.751304

**Published:** 2021-11-23

**Authors:** Cellas A. Hayes, Brandon G. Ashmore, Akshaya Vijayasankar, Jessica P. Marshall, Nicole M. Ashpole

**Affiliations:** ^1^Department of BioMolecular Sciences, University of Mississippi School of Pharmacy, University of Mississippi, Oxford, MS, United States; ^2^Research Institute of Pharmaceutical Sciences, University of Mississippi School of Pharmacy, University of Mississippi, Oxford, MS, United States

**Keywords:** astrocytes, endothelial cells, neurotoxicity, somatomedin C, reactive oxygen species

## Abstract

The age-related reduction in circulating levels of insulin-like growth factor-1 (IGF-1) is associated with increased risk of stroke and neurodegenerative diseases in advanced age. Numerous reports highlight behavioral and physiological deficits in blood-brain barrier function and neurovascular communication when IGF-1 levels are low. Administration of exogenous IGF-1 reduces the extent of tissue damage and sensorimotor deficits in animal models of ischemic stroke, highlighting the critical role of IGF-1 as a regulator of neurovascular health. The beneficial effects of IGF-1 in the nervous system are often attributed to direct actions on neurons; however, glial cells and the cerebrovasculature are also modulated by IGF-1, and systemic reductions in circulating IGF-1 likely influence the viability and function of the entire neuro-glio-vascular unit. We recently observed that reduced IGF-1 led to impaired glutamate handling in astrocytes. Considering glutamate excitotoxicity is one of the main drivers of neurodegeneration following ischemic stroke, the age-related loss of IGF-1 may also compromise neural function indirectly by altering the function of supporting glia and vasculature. In this study, we assess and compare the effects of IGF-1 signaling on glutamate-induced toxicity and reactive oxygen species (ROS)-produced oxidative stress in primary neuron, astrocyte, and brain microvascular endothelial cell cultures. Our findings verify that neurons are highly susceptible to excitotoxicity, in comparison to astrocytes or endothelial cells, and that a prolonged reduction in IGFR activation increases the extent of toxicity. Moreover, prolonged IGFR inhibition increased the susceptibility of astrocytes to glutamate-induced toxicity and lessened their ability to protect neurons from excitotoxicity. Thus, IGF-1 promotes neuronal survival by acting directly on neurons and indirectly on astrocytes. Despite increased resistance to excitotoxic death, both astrocytes and cerebrovascular endothelial cells exhibit acute increases in glutamate-induced ROS production and mitochondrial dysfunction when IGFR is inhibited at the time of glutamate stimulation. Together these data highlight that each cell type within the neuro-glio-vascular unit differentially responds to stress when IGF-1 signaling was impaired. Therefore, the reductions in circulating IGF-1 observed in advanced age are likely detrimental to the health and function of the entire neuro-glio-vascular unit.

## Introduction

Aging is a primary risk factor for a number of diseases and pathologies, including stroke, which is recognized as one of the leading causes of death and disability globally (Johnson et al., 2016; Benjamin et al., 2019). Ischemic stroke is particularly devastating as the long-term phenotypic deficits in cognitive behavior and sensorimotor function build in the days following the insult, even if blood flow to the ischemic tissue is restored [as reviewed by George and Steinberg (2015)]. Ischemia precipitates cellular damage in the brain within minutes, as the loss of blood flow induces metabolic and oxidative stress in the energy-demanding neurons, and nearby glial and cerebrovascular cells. Immediate restoration of blood flow is critical, as the length of ischemia correlates with the extent of tissue damage (Terasaki et al., 2014). To date, the most effective ischemic stroke therapy is fibrinolytic breakdown of the clot with administration of tissue plasminogen activator. Unfortunately, this therapy is time-restricted and requires confirmation of ischemic vs hemorrhagic manifestation, which restricts clinical utility and has limited its use to around 5% of stroke patients each year (National Institute of Neurological Disorders and Stroke rt-Pa Stroke Study Group, 1995). Longitudinal observation-based clinical assessments have identified multiple possible biomarkers of stroke risk and severity, including insulin-like growth factor-1 (IGF-1), which may serve as a point of therapeutic intervention in the days surrounding insult (Katzan et al., 2000; California Acute Stroke Pilot Registry Investigators, 2005; Qureshi et al., 2005; Kleindorfer et al., 2008).

Insulin-like growth factor-1 is a pleiotropic neuroendocrine modulator that has been assessed in both clinical and preclinical studies of stroke [as reviewed by Hayes et al. (2021)]. Both humans and rodents exhibit age-related circulating declines of IGF-1 (O’Connor et al., 1998; Sonntag et al., 2013). IGF-1 levels decline in advanced age, and the extent of this decline is associated with increased risk of stroke incidence, increased mortality, and worsened functional outcomes post-stroke in humans (Johnsen et al., 2004; De Smedt et al., 2011; Tang et al., 2014; Armbrust et al., 2017; Saber et al., 2017). Moreover, altered IGF-1 levels are also associated with increased risk of multiple age-related neuropathologies marked by neurovascular distress, excitotoxicity, and oxidative stress (Watanabe et al., 2005; Sonntag et al., 2013; Ghazi Sherbaf et al., 2018; Gubbi et al., 2018). While many studies associate reduced IGF-1 with increased neurodegeneration, some clinical studies of dementias and stroke see increased IGF-1 levels at the start of pathology, which may be an attempted compensatory mechanism designed to protect the brain (Gubbi et al., 2018; Castilla-Cortazar et al., 2020). Preclinical studies suggest that IGF-1 plays a causal role in the stroke risk and recovery processes by directly regulating cell damage during ischemia [as reviewed by Hayes et al. (2021)]. IGF-1 supplementation in rodent models of middle cerebral artery occlusion and photothrombotic stroke reduces the size of infarcted tissue and the accompanying sensorimotor deficits, even when administered in the days after insult (Liu et al., 2001; Bake et al., 2014, 2016; Parker et al., 2017; Serhan et al., 2019; Hayes et al., 2021). Because IGF-1 regulates numerous cells throughout the body, the cellular mechanism by which IGF-1 protects viability and function in the brain remains unclear. It is commonly inferred that the beneficial effects of IGF-1 are mediated by direct regulation of neurons. However, neurons, astrocytes, endothelial cells, and perivascular cells each express the receptor for IGF-1, IGFR. Together, these cells work to compose the neurovascular unit (also known as neuro-glio-vascular unit) which tightly coordinates physiological responses within the nervous system and is a central target for dysfunction in neurodegenerative diseases and pathologies (Stanimirovic and Friedman, 2012). Additional work is needed to tease apart the impact of each cell type when exogenous IGF-1 is administered to protect against the damage induced by stroke and neurodegenerative diseases.

Animal models of circulating IGF-1 deficiency exhibit impaired neurovascular coupling, compromised blood-brain barrier integrity, and increased prevalence of micro hemorrhages (Toth et al., 2015; Tarantini et al., 2016b, 2017, 2021a; Hayes et al., 2021). Increased reactive oxygen species (ROS) production, increased inflammation, and reduced glutamate and glucose handling machinery accompany the loss of circulating IGF-1 in these models (Toth et al., 2015, 2017). Together, these data suggest that the age-related reduction in IGF-1 likely leads to severe consequences on the structure and function of the neuro-glio-vascular unit. Indeed, *in vitro* studies have highlighted roles for IGF-1 in promoting specific functions of each cellular component of the neuro-glio-vascular unit. Pharmacological and genetic manipulations of IGF-1 alter the viability and oxidative stress levels of cultured neurons (Wang et al., 2014; Li et al., 2017; Chen et al., 2019). Additionally, IGF-1 signaling in brain vascular endothelial cell cultures promotes angiogenesis and tight-junction formation/integrity (Lopez-Lopez et al., 2004; Bake et al., 2016; Higashi et al., 2020). Genetic reductions of IGFR specifically in endothelial cells impairs stimulation-induced cerebral blood flow, indicating that the loss of IGF-1 signaling in one component of the neurovascular unit results in phenotypic changes commonly observed in the aged brain (Tarantini et al., 2021b). We recently reported that deficiency in IGF-1 signaling in astrocytes impairs glutamate handling *in vitro* and *in vivo*, by reducing glutamate transporter expression and availability at the cell surface (Prabhu et al., 2019). Although there are thought to several key drivers to aging pathologies and disease, glutamate over excitation is a central contributor to tissue damage following ischemic stroke (Choi and Rothman, 1990; Moskowitz et al., 2010), it is likely that the age-related loss of IGF-1 may compromise the neuro-glio-vascular unit by weakening the glutamate-buffering capabilities of astrocytes which ultimately exacerbates calcium imbalances, mitochondrial dysfunction, and oxidative stress. While the aforementioned studies highlight the ability of IGF-1 to regulate neurons, astrocytes, and brain-derived endothelial cells, each of these studies was performed under varied conditions. A comparative approach is needed to better-understand how the age-related loss of IGF-1 influences cells within the neuro-glio-vascular unit. Thus, we exposed individual cell cultures and co-cultures to excitotoxic levels of glutamate when IGF-1 signaling was impaired. This approach allows for determination of the cellular mechanism(s) by which IGF-1 exerts its protective effects during ischemic stroke-induced stressors. In addition, it highlights cell-specific responses to a common driver of neuronal dysfunction- glutamate excitotoxicity- which has applications to numerous age-related neurodegenerative disease states.

## Materials and Methods

### Animals

All procedures were approved by the Institutional Animal Use and Care Committees of the University of Mississippi, and performed in accordance with their approved guidelines. Timed-pregnant Sprague Dawley rats (16–18 days postictal plug) were purchased (Envigo) and were temporarily housed in 19 × 11.5 × 11 inch polycarbonate cages until the time of euthanasia. Rats were given access to standard rat chow (Teklad 7001) and water *ad libitum.* At the time of euthanasia, pregnant dams were anesthetized with isoflurane and cervical dislocation was completed. Embryonic pups (E17–19) were excised and rapidly decapitated. Due to the non-proliferative nature of neurons, the number of cells needed seeded per well, and the replication of results across multiple primary cell culture preparations, multiple pregnant dams were needed for these studies (approximately 7). The average litter size of a Sprague Dawley female is 10–11 pups, and cerebral tissues of all pups were pooled at the time of euthanasia. Isolated embryonic rat pups were not separated based on sex; thus, our studies are indicative of cell responses from both sexes combined. To reduce animal number, astrocytes and neurons were both cultured from the pooled tissues at the same time, in varying selection medias, as described below.

### Neuron Cultures

Primary rat neuron cultures were established following previously described protocols (Ashpole and Hudmon, 2011). Cell culture dishes and coverslips were coated with Poly-D-Lysine (PDL; Sigma P6407) for at least 2 h at 37°C in a humidified incubator containing 5% CO2. Individual cultures were plated in 96-well plates, while 15 mm glass coverslips were used to plate neurons for the triple co-cultures. Following PDL coating, cell culture surfaces were washed with 1X PBS (Gibco 10010-023) and dried. Primary neuronal cultures were derived from the cortex and hippocampus of E17-19 Sprague Dawley rat pups. External cerebrovasculature was removed, the tissue was minced and subsequently enzymatically and mechanically digested with papain (Worthington Biochemical Corporation, LS003126) and glass-blown pipettes, respectively. Dissociated cells were pelleted with centrifugation (500–1,000 *g*, 5 min) and suspended in neuron growth media [Neurobasal medium (Gibco 21103-049) containing B27 (Life Technologies, 17504044), penicillin/streptomycin (10 units/mL; Life Technologies, 15140122), and 1x L−glutamine (250303-081)] with a target density of 2,500,000/mL. As neurons are non-dividing, the density of cells plated is indicative of the density at the time of experimentation; less than 10% of the cells in these cultures are GFAP + or IBA + positive, indicating they are predominantly neurons (Ashpole and Hudmon, 2011). Partial media changes occurred every 3–4 days post seeding to replenish nutrients and remove debris.

### Astrocyte Cultures

Tissue from male and female rat pups was isolated, digested, and pelleted following the methodology described above for neuron cultures, and neurons and astrocytes were derived from the same isolated tissues. To promote astrocyte selection after digested tissue was centrifuged, cell pellets were suspended in astrocyte growth media containing Neurobasal medium (Gibco 21103-049) with 10% fetal bovine serum (Corning 35-010-CV), penicillin/streptomycin (10 units/mL; Life Technologies, 15140122), and 1x L−glutamine (250303-081). Cells were plated on PDL-coated 10 cm dishes and media was completely exchanged the day following plating. To further select for astrocytes, the cells were sub-cultured every 3–4 days (90–100% confluent). For this, the growth media was removed, cells were washed with 1X PBS, and 0.05–0.25% of Trypsin-EDTA 1X (Gibco 25200-072) was added. To avoid excess debris, trypsin was applied twice during the first passage- once for 2 min to dislodge neurons and microglial cells from the upper layer (vacuumed off), and a second time to release the astrocytes underneath. Once the astrocytes dislodged, the cells were centrifuged (1,000 × *g*; 5 min) and suspended in growth medium for further plating. All astrocyte cultures were passaged multiple times prior to experimentation (as described above), and the growth rate was continuously monitored to determine if cells were exhibiting replicative senescence. Cells were not passaged more than 6 times to avoid confounds of senescent phenotypes. The resulting cultures are >90% GFAP+, indicating they are astrocytes (Prabhu et al., 2019).

### Endothelial Cell Culture

Rat brain microvascular endothelial cells (RBMVECs) and medium were purchased from Cell Applications Inc. (San Diego) and cultured following the manufacturer’s guidelines. A single vial of RBMVEC were used to conduct all included studies. RMBVEC were continually passaged and 20% of cells were frozen at each passage throughout the study for replicate, follow-up, and future studies to reduce variance from a different lot of cells originally isolated. In brief, cells were grown on a T-75 flasks pre-coated with Cell Attachment Factor Solution (123-100, Cell Applications), in 15 mL of RBMVEC growth medium (R819-500). Growth medium was changed 24 h after initial seeding, and cells were sub-cultured every 4–6 days or when at 80–100% confluency. Media was changed every other day. RBMVECs were passaged using the same protocol as astrocytes, using the proprietary trypsin solution (Cell Applications Inc).

### Co-cultures

Triple cultures were developed by plating primary neurons on glass coverslips, RBMVECs on transwell inserts, and astrocytes on the surface of a multi-well plate. Astrocytes were cultured and passaged at least 1 week prior to combination with the other cells, to allow for selection of astrocytes away from neurons and microglial cells. Neurons were also cultured 7–8 days prior to combination and experimentation, to allow for development/expression of ionotropic glutamate receptors (GluNR) responsible for excitotoxic signaling. Coverslips were elevated off the surface of the multi-well plate and astrocytes were seated in the outer 1/3 of the wells. The media on all 3 cell types was exchanged for neuron growth media 24 h prior to combination of the co-cultures to allow for uniformity between media types.

### Cells Treatments

Glutamate stimulation was performed using L-glutamic acid and glycine (FisherScientific) in a ratio of 10:1 glutamate:glycine diluted from a PBS stock solution. Increasing concentrations of glutamate were prepared in the appropriate cell growth media, and administered as a 2x solution to the cells plated in their respective media (final concentration 1x upon application). Cells were incubated for 1 h at 37°C. Following treatment, glutamate media was removed, cells were washed in fresh growth media twice, and incubated in new growth media until the designated time point. All cell viability studies were conducted 24 h after cells were stimulated with glutamate. All other study time points are described in the respective sections. In one experimental series, media lacking the B-27 supplement was administered 24 h prior to glutamate stimulation.

Stock solutions of picropodophyllin toxin (PPP; Sigma T-9576) and MK-801 (Sigma M107) were prepared in dimethyl sulfoxide (DMSO; Fisher BP321-100) and administered to cells at concentrations and time-points described. Treatment groups were block randomized to ensure equal sampling. Individual wells in the multi-well plates were treated as independent samples; and assays with neurons and astrocytes were further repeated across multiple tissue preparations to verify reproducibility of findings in cells isolated from separate rat pup litters.

### Cell Viability

Following treatments, cell viability/cytotoxicity was quantified using Live:Dead viability/cytotoxicity kit (Thermo Fisher Scientific L3224). Calcien-AM and ethidium homodimer were diluted (1:1000-2000; 1:500-1000) were diluted in PBS. Growth media was removed and cells were washed with 1X PBS prior to adding the live/dead labels. After 20 min of incubation, cells were imaged on the Nikon Ti2-E HCA inverted fluorescent microscope using the JOBS automated image acquisition (Nikon). Magnification was set to 200x (20x, extra-long working distance objective) and samples were excited with the LED Triggered acquisition exposures using excitation/emission filters for 470-FITC and 540-TRITC. The JOBS program selected 6 fields at random per well for imaging, and the total number of live and dead cells were quantified in 2–3 images using the Nikon Elements Cell Analysis plug-in features. The total number of cells and percent toxicity were calculated by observers blinded to treatment groups.

### Reactive Oxygen Species Quantification

Following treatment, ROS production was quantified in astrocytes and endothelial cells. Media was removed, and cells were washed with 1X PBS. Cells were then loaded with 3 μM of 2′,7′-dichlorodihydrofluorescein diacetate (DCFDA; Thermofisher, D399) for 20 min and incubated at 37°C in a humidified incubator containing 5% CO^2^. DCFDA was then imaged using the FITC filter on the Nikon Ti2-E microscope as described above. Six images at random through the JOBS interface and 4–6 non-adjacent cells per field were selected at random to be quantified by a blinded observer. DCFDA intensity of each cell was normalized to the background of that image.

### Seahorse

Cells were seeded on the XFe96 growth plates 2–3 days prior to extracellular efflux assessment. Treatments were administered at the desired time points and mitochondrial response was assessed using the Cell Mito Stress Test, per manufacturer recommendations. The sensor cartridge was hydrated in water overnight at 37°C and then equilibrated with the recommended equilibration buffer. Oligomycin (1.5 μM), FCCP (1 μM), and rotenone (0.5 μM) were loaded into the sensor plates and injected into the wells by the XFe/XF Seahorse system. Basal and maximal respiration, as well as proton leak were auto-calculated by the Seahorse program using an average of 3 readings per stage.

### Data Analysis

Statistical analysis and graphical analyses were performed using Sigma Plot version 14 software and R studio. One-way and two-way ANOVAs was used when appropriate (defined in the figure legend). *Post hoc* comparisons were selected based upon the experimental question and fulfillment of normality and equal distribution. Details for each are provided in figure legend. All data were expressed with mean ± standard error. Sample sizes were estimated using previous variance observations with these experimental methodologies. With β = 0.8, *n* = 4–6 was required to achieve power for most studies. *F* values for two-way ANOVAs are described in the results and main effects are noted in figures with a pound sign (#). For all studies, *p* < 0.05 was used as the statistical significance value and denoted using an asterisk (^∗^).

## Results

### Insulin-Like Growth Factor-1 Protects Against Excitotoxicity in Neurons

**FIGURE 1:**
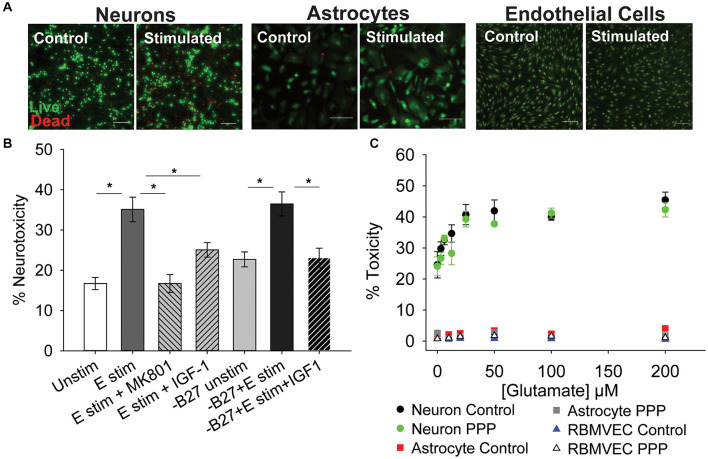
IGF-1 protects against excitotoxicity in glutamate-sensitive neurons. **(A)** Representative images of neurons (left two panels), astrocytes (middle two panels), and rat brain microvascular endothelial cells (right two panels) stained with live (green)/Dead (red) viability/cytotoxicity indicator dyes 24 h after stimulation with control or 100 μM glutamate, as indicated. Scale bar represents 100 μm. **(B)** Average toxicity in neurons 24 h after treatment (*n* = 8 wells/group). A one-way ANOVA with *post hoc* Bonferroni was used for statistical comparisons; *indicates significant difference between the delineated bars. **(C)** Average toxicity of cells 24 h after acute treatment with both 0.5 μM PPP and increasing concentrations of glutamate (*n* = 4–8 wells/group). A two-way ANOVA was employed with glutamate concentrations and PPP-treatment serving as factors. No significant differences were observed. Graph color code: black circles = vehicle-treated neurons; green circles = PPP-treated neurons; red boxes = vehicle-treated astrocytes; dark gray boxes = PPP-treated astrocytes; blue triangles = vehicle-treated rat brain microvascular endothelial cells (RBMVEC); and light gray triangles = PPP-treated RBMVEC. All data are presented as mean ± SEM.

To determine if inhibition of IGF-1 signaling differentially affects the glutamate sensitivity of cells within the neuro-glio-vascular unit, cultures of neurons, astrocytes, and microvascular endothelial cells were established. Primary cerebral neurons were isolated from embryonic rat pups (male and female combined) and grown in culture for 8–10 days. Primary neurons at this stage of development are sensitive to excitotoxicity when exposed to high concentrations of glutamate and glycine, due to overactivation of ionotropic GluNR. As expected, acute treatment of neurons with 100 μM glutamate/10 μM glycine (termed glutamate stimulation throughout) resulted in a significant increase in cellular toxicity 24 h after stimulation (Figures 1A,B; *p* < 0.001 vs unstimulated). Co-administration of the GluNR antagonist MK-801 prevented excitotoxicity (Figure 1B; *p* < 0.001 vs glutamate stimulated control). Administration of exogenous 100 nM IGF-1 at the time of stimulation also prevented excitotoxicity (*p* = 0.019 vs glutamate stimulated control), consistent with previous reports. No differences in the total number of neurons per field were noted following treatment (Supplementary Figure 1A).

The neuronal growth media contains a supplement (B27) that has a high concentration of insulin, which can cross-talk and activate IGFR. Thus, to better determine the protective effects of IGF-1 signaling, the excitotoxicity profile was again assessed without B27 present. No change in baseline levels of toxicity were observed between control neurons with and without B27, and glutamate stimulation still significantly increased toxicity in the absence of B27 (Figure 1B; *p* = 0.002 vs –B27 unstimulated control). Once more, exogenous IGF-1 prevented excitotoxicity (Figure 1B; *p* = 0.003 vs –B27 glutamate stimulated control).

To more specifically assess the impact of reducing IGF-1 signaling, the extent of excitotoxicity when IGFR was pharmacologically inhibited was then examined. For this, picropodophyllotoxin (PPP), a known small molecule inhibitor of the IGFR receptor kinase activity, was co-administered at the time of glutamate stimulation and toxicity was measured 24 h later. Surprisingly, PPP did not shift the concentration-dependence or maximal extent of glutamate toxicity in cultured neurons (Figure 1C). A two-way ANOVA revealed a significant effect of glutamate concentration (*F* = 13.759, *p* < 0.001), but no significant effect of PPP treatment (*F* = 2.598, *p* = 0.111), and no significant interaction between the two factors (*F* = 0.506, *p* = 0.827).

Astrocytes and microvascular endothelial cells are not as sensitive to glutamate as neurons as no change in toxicity was observed across all glutamate concentrations (Figures 1A–C). Similarly, no increase in sensitivity was observed with IGFR inhibition at the time of glutamate stimulation (Figure 1C).

### Prolonged IGFR Inhibition Increases Astrocytic Sensitivity to Glutamate

**FIGURE 2:**
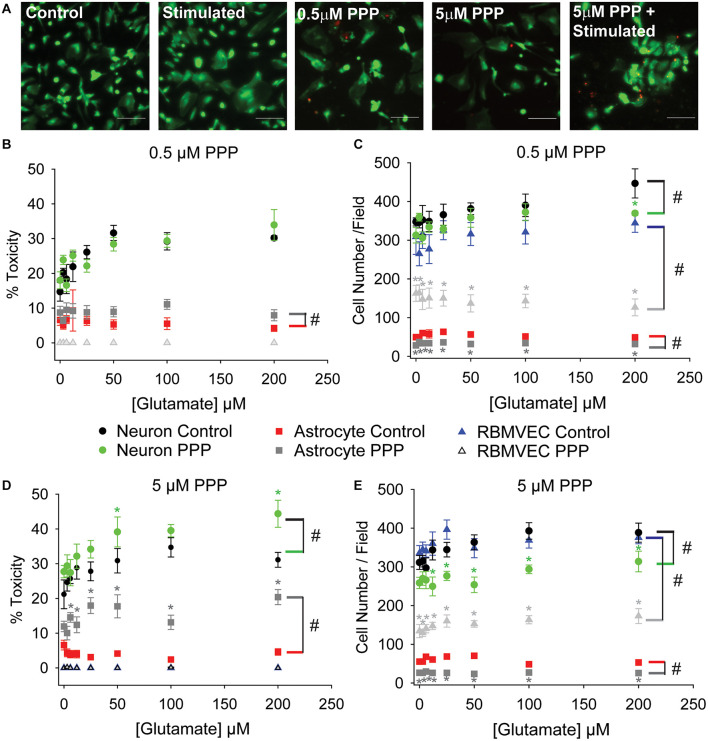
Prolonged IGFR inhibition increases astrocytic sensitivity to glutamate and reduces astrocyte and endothelial cell number. **(A)** Representative images of astrocytes stained with live (green)/Dead (red) viability/cytotoxicity indicator dyes. From left to right: unstimulated control, glutamate-stimulated vehicle-treated, unstimulated 0.5 μM PPP-treated, unstimulated 5 μM PPP, and glutamate-stimulated 5 μM PPP. Scale bar represents 100 μm. **(B)** Average toxicity and **(C)** average cell number per well of cells pre-treated with 0.5 μM PPP, and subsequently stimulated with increasing concentrations of glutamate, 24 h after stimulation. **(D)** Average toxicity and **(E)** average cell number per well of cells pre-treated with 5 μM PPP, and subsequently stimulated with increasing concentrations of glutamate, 24 h after stimulation. All data are presented as mean ± SEM, *n* = 5–8 wells/treatment group. A two-way ANOVA was employed with glutamate concentrations and PPP-treatment serving as factors. Cell types were each analyzed independently. ^#^ indicates a significant main effect within that cell type. *Post hoc* Holm-Sidak tests were used for pairwise multiple comparisons. *indicates a significant difference between PPP-treatment and vehicle-treatment within a given glutamate concentration. Graph color code: black circles = vehicle-treated neurons; green circles = PPP-treated neurons; red boxes = vehicle-treated astrocytes; dark gray boxes = PPP-treated astrocytes; blue triangles = vehicle-treated rat brain microvascular endothelial cells (RBMVEC); and light gray triangles = PPP-treated RBMVEC.

The lack of effect with pharmacological inhibition of IGFR in Figure 1C was inconsistent with the protective effects of exogenous IGF-1 in Figure 1B. However, co-administration of PPP at the time of glutamate could have temporal confounds that limit interpretation. Thus, we next administered PPP 24 h prior to glutamate stimulation to better mimic the prolonged loss of IGFR signaling observed in aging. Prolonged IGFR inhibition with 0.5 μM PPP did not lead to difference in neuronal excitotoxicity (*F* = 0.255, *p* = 0.615), but did lead to differences in astrocyte viability (*F* = 6.677, *p* = 0.011; Figures 2A,B). Within group comparisons did not unveil a specific concentration of glutamate that PPP treatment significantly worsened, thus it is assumed that IGFR inhibition led to modest reductions in cell viability across all glutamate treatments.

No changes in endothelial cell viability were observed with prolonged IGFR inhibition; however, a pronounced reduction in total cell number was noted when microvascular endothelial cells were treated with 0.5 μM PPP (*F* = 117.942, *p* < 0.001; Figure 2C). A significant difference within unstimulated controls with PPP pre-treatment was detected (*p* < 0.001), and this effect persisted across all glutamate concentrations. Thus, no main effect of, or interaction with, glutamate concentration was observed in the treated microvascular endothelial cells.

The change in endothelial cell number prompted further analyses of neuron and astrocyte counts with treatment. Similar reductions in astrocyte count were observed with 0.5 μM PPP treatment (*F* = 73.322, *p* < 0.001; Figures 2A–C). Glutamate concentration did not alter astrocyte count nor interact with the PPP effect. Main effects of both glutamate concentration and PPP treatment on total neuron count were detected (*F* = 3.309, *p* = 0.004; *F* = 7.835, *p* = 0.006, respectively). *Post hoc* comparisons within groups revealed that the only significant difference within the PPP vs vehicle treatments was the drop in total cell number at 200 μM glutamate (*p* = 0.012), likely an indication of dead cells washed away during the staining process at this high concentration of glutamate.

When the concentration of PPP was increased to 5 μM, significant differences in neuron and astrocyte survival were observed (Figure 2D). Both glutamate concentration and PPP treatment had significant main effects on neurotoxicity (*F* = 4.272, *p* < 0.001; *F* = 12.565, *p* < 0.001, respectively). However, no interaction between the two factors was detected (*F* = 0.732; *p* = 0.646). Within group comparisons revealed significant increases in neurotoxicity when neurons pre-treated with 5 μM PPP were stimulated with 50 and 200 μM glutamate (*p* = 0.048 and *p* = 0.006, respectively). No baseline differences in toxicity amongst unstimulated controls treated with vehicle or PPP were observed (*p* = 0.751). A significant reduction in total neuron number was observed with 5 μM PPP treatment and with glutamate (*F* = 49.058, *p* < 0.001; *F* = 2.878, *p* = 0.009, respectively; Figure 2E). No significant interactions between the factors was detected (*F* = 0.932, *p* = 0.485). Within factor comparisons showed decreased cell number within PPP-treated neuron cultures stimulated with ≥12.5 μM glutamate, again suggesting potential loss of dead cells during the staining process since neurons are non-mitotic.

Prolonged IGFR inhibition in astrocytes also resulted in main effects of glutamate concentration and PPP treatment on the levels of cytotoxicity (*F* = 157.321, *p* < 0.001; *F* = 2.209, *p* = 0.039, respectively; Figure 2D). Moreover, a significant interaction between glutamate concentration and PPP treatment was also observed (*F* = 2.743, *p* = 0.011). Within factor comparisons revealed significant increases in PPP-pretreated astrocytes stimulated with ≥6 μM glutamate, suggesting that while astrocytes display increased resistance to glutamate toxicity under control conditions, the loss of IGF-1 signaling predisposes astrocytes to excitotoxic stress. A significant reduction in astrocyte cell number was also observed with 5 μM PPP treatment (*F* = 257.893, *p* < 0.001), across all glutamate concentrations. Main effect differences for glutamate concentration did not reach significance (*F* = 1.882, *p* = 0.079), nor did interactions between factors (*F* = 2.071, *p* = 0.052). Prolonged treatment with another IGFR inhibitor, NCP-ADW742, also increased susceptibility to glutamate excitotoxicity and reduced astrocyte cell number (Supplementary Figures 1B,C). Baseline toxicity levels and cell numbers were observed with administration of supplemental IGF-1 prior to glutamate stress (Supplementary Figures 1B–E), indicating that the prolonged loss of IGF-1 predisposes astrocytes to glutamate toxicity, despite their typical resistance to the stress.

Similar to the before no differences in endothelial cell toxicity were observed across any treatment groups, but 5 μM PPP did reduce cell number across all glutamate concentrations (*F* = 538.084, *p* < 0.001; Figure 2E). Again, this suggests that while IGFR inhibition did not predispose endothelial cells to glutamate toxicity, it likely reduces cell division.

### Astrocytes Fail to Protect Neurons When IGFR Is Inhibited

**FIGURE 3:**
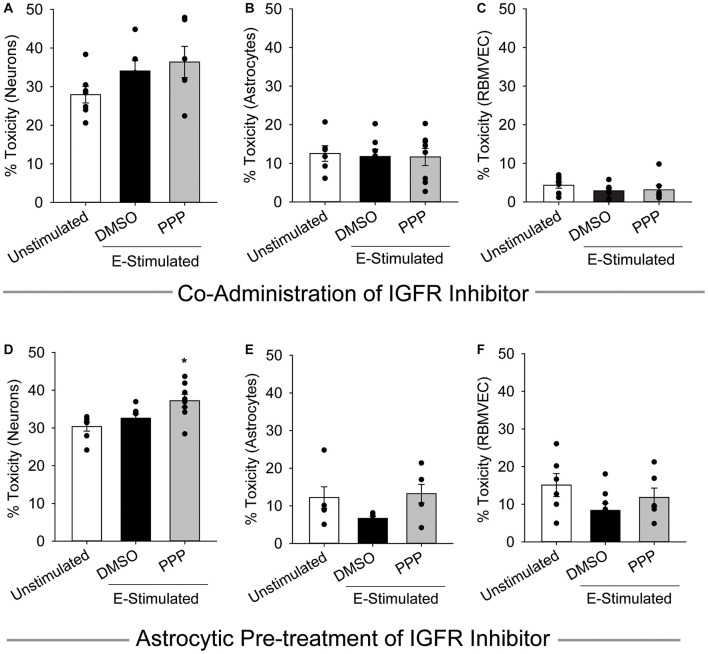
Astrocytes fail to protect against excitotoxicity when IGFR is inhibited. Average cell death 24 h after triple cultures of neurons **(A)**, astrocytes **(B)**, and endothelial cells **(C)** were co-administered 0.5 μM PPP and 100 μM glutamate for 1 h (*n* = 6–8 wells/group). One-way ANOVA failed to detect differences in any of the treatment groups. **(D–F)** Astrocytes were pre-treated with 5 μM PPP for 24 h prior to combining with endothelial cells and neurons for a triple culture system. Average cell death in the neurons **(D)**, astrocytes **(E)**, and endothelial cells **(F)** after stimulation with 100 μM glutamate for 1 h (*n* = 5–8 wells/group). One-way ANOVAs with *post hoc* Bonferroni comparison was used for statistical analyses. *indicates significant difference between control and PPP-treated glutamate-stimulated neurons. All data are presented as mean ± SEM, *n* = 5–8 wells/treatment group.

Cells within the neuro-glio-vascular unit coordinate to create an optimal microenvironment, by releasing growth mediators, buffering stressors, and regulating nutrient supply. Thus, we next assessed whether co-cultures of endothelial cells, neurons, and astrocytes would exhibit differences in glutamate sensitivity when IGFR is inhibited. As anticipated, 100 μM glutamate did not significantly increase neurotoxicity in the presence of astrocytes and endothelial cells, nor did acute co-administration of 0.5 μM PPP with 100 μM glutamate (*p* = 0.139; Figure 3A). No differences in astrocyte or endothelial cell toxicity were observed in these acute treatments (Figures 3B,C).

When astrocytes alone were pre-treated with 5 μM PPP and then combined with neurons and endothelial cells for glutamate stimulation, a significant increase in neurotoxicity was observed (*p* = 0.007 vs control) (Figure 3D). No differences in toxicity or cell number were noted in either the astrocytes or endothelial cells within the triple cultures (Figures 3E,F and Supplementary Figures 1D–F). Together these data suggest that a reduction of IGFR signaling in astrocytes impairs their ability to buffer glutamate and protect neurons from overexcitation, even when in the presence of endothelial cells.

### Astrocytic and Endothelial Reactive Oxygen Species Levels Are Elevated by IGFR Inhibition and Glutamate Stimulation

**FIGURE 4:**
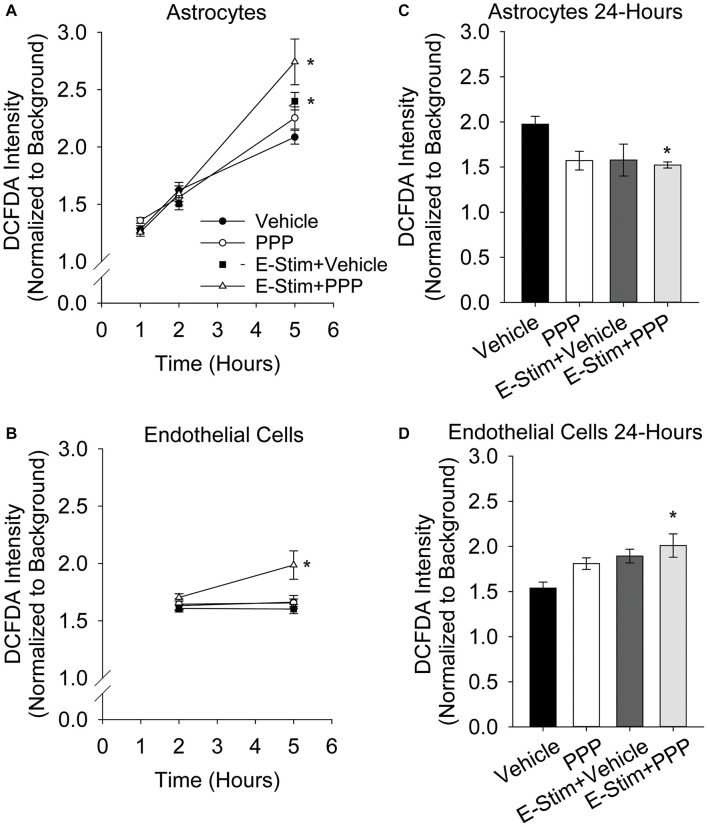
Astrocytic and endothelial ROS levels are elevated by IGFR inhibition and glutamate stimulation. Average ROS levels in pure cultures of astrocytes **(A)** or endothelial cells **(B)** co-treated with 0.5 μM PPP and 100 μM glutamate for 1 h, and subsequently measured 1, 2, and 5 h after treatment. Four cells were selected at random per field, with 3 fields per well, and 8–10 wells per treatment group. Background intensity within each image was measured and used to normalize. A two-way ANOVA was employed with both time and treatment as factors, and a *post hoc* Holm-Sidak was used to compare within groups. * indicates a significant effect of treatment at that time. Average ROS levels in pure cultures of astrocytes **(C)** or endothelial cells **(D)** co-treated with 0.5 μM PPP and 100 μM glutamate for 1 h, and subsequently measured 24 h after treatment. Four cells were selected at random per field, with 3 fields per well, and 4–8 wells per treatment group. A one-way ANOVA with *post hoc* Dunnett’s test was used for statistical comparisons to control. * indicates a significant effect vs control. All data are presented as mean ± SEM.

While acute co-administration of 0.5 μM PPP and glutamate did not increase toxicity in astrocyte or endothelial cultures, alterations in metabolic function and oxidative stress may still occur with this stressor. Thus, astrocytes and endothelial cells were treated with 100 μM glutamate alone, or in conjunction with 0.5 μM PPP, and the production of ROS was quantified at various time points following treatment. Astrocytic ROS production increases in the hours following glutamate stimulation (Figure 4A). Significant effects of both time and treatment were observed (*F* = 185.57, *p* < 0.001; *F* = 3.187, *p* = 0.027, respectively), as well as an interaction between both factors (*F* = 4.586, *p* < 0.001). 5 h post-treatment, vehicle treated and PPP-treated astrocytes stimulated with glutamate showed significant increases in ROS levels over unstimulated controls (both *p* < 0.001 vs unstimulated control). Moreover, co-administration of PPP and glutamate resulted in significantly increased ROS levels than glutamate stimulated controls (*p* = 0.014), suggesting that the extent of glutamate-induced ROS production was exacerbated by IGFR inhibition. 5 h after glutamate stimulation with another IGFR inhibitor, NVP-ADW742, also significantly increased ROS (Supplementary Figure 2A), indicating the effect is not limited to PPP. Additional analysis of mitochondrial bioenergetics at this time point revealed that glutamate stimulation decreased maximal extracellular acidification rates, while PPP + glutamate decreased extracellular acidification and oxygen consumption rates, suggesting changes in both glycolysis and oxidative phosphorylation (Supplementary Figure 2B). No differences in basal respiration rates were noted with any of the treatments (Supplementary Figure 2B).

Similar to astrocytes, brain-derived microvascular endothelial cells showed increased ROS production 5 h after 0.5 μM PPP was co-administered with 100 μM glutamate (Figure 4B). A main effect treatment was observed (*F* = 5.287, *p* = 0.004). While glutamate alone did not increase ROS production in the endothelial cells (*p* = 0.945), a significant increase in ROS was seen in cells treated with PPP + glutamate vs unstimulated control (*p* = 0.02), and vs glutamate-stimulated control (*p* = 0.004).

Additional analyses of ROS levels 24 h following treatment revealed differential recovery from acute IGFR inhibition and glutamate stimulation. Astrocytes do not show increased ROS at 24 h with 100 μM glutamate or 100 μM glutamate + 0.5 μM PPP treatment (Figure 4C). In fact, ROS levels were significantly reduced in glutamate-stimulated PPP-treated astrocytes vs unstimulated controls (*p* = 0.024) and PPP treatment was trending (*p* = 0.053 vs control). Endothelial cells continue to show increased ROS levels long after the acute exposure to 100 μM glutamate + 0.5 μM PPP (*p* = 0.013 vs unstimulated control; Figure 4D), suggesting that IGFR inhibition at the time of glutamate stimulation increases endothelial ROS levels long-term. Note, this was with acute PPP treatment, at a time point in which no differences in endothelial cell toxicity was observed (Figure 1C).

## Discussion

The age-related loss of IGF-1 has been linked to cognitive impairment, neurodegeneration, and increased susceptibility ischemic stroke and other neurovascular pathologies (Sonntag et al., 2013; Ashpole et al., 2015b). These deficits often come as a disconnect from the enhanced longevity observed in animal models of IGF-1 deficiency. While early-life reductions in circulating IGF-1 do lead to increased lifespan, reduced IGF-1 in advanced age has multiple consequences in animal models as it increases sarcopenia, bone frailty, learning and memory deficits, and cerebrovascular dysfunction (Ashpole et al., 2015a, 2016, 2017; Toth et al., 2015; Tarantini et al., 2016a, 2021a; Farias Quipildor et al., 2019). For example, IGF-1 deficiency in mice decreases stimulation-evoked cerebral blood flow, blood brain barrier integrity, and the strength and flexibility of cerebral arteries. IGF-1 deficiency also impairs the production and release of vasomediator eicosanoids from astrocytes, alters endothelial nitric oxide production, and increases susceptibility to hypertension-induced microhemorrhages (Toth et al., 2015; Tarantini et al., 2017, 2021a; Fulop et al., 2019). Considering this, it is not surprising that IGF-1 has long-been implicated in the risk and severity of ischemic stroke. As described earlier, clinical and preclinical evidence highlight inverse correlation (and causation in animal models) between IGF-1 and the risk/outcome of ischemic stroke (Hayes et al., 2021).

While recent studies are beginning to shed light on the impact of IGF-1 on neurovascular communication in advanced age, there remains a significant gap in our understanding of how individual cell types that normally coordinate signaling together within the neurovascular unit each respond to reduced IGF-1 signaling. In this study, we aimed to compare the effects of IGF-1 deficiency on the cellular responses to high levels of extracellular glutamate. As the predominant excitatory neurotransmitter in the brain, glutamate induces depolarization and initiates multiple calcium signaling cascades. During periods of ischemia or proteotoxic stress, neurons experience energy imbalances and ionic stressors that ultimately lead to terminal depolarization and the dumping of glutamate stores, inflammatory mediators, and oxidative stressors into the extracellular space, which expands the region of distress and malfunction [as recently reviewed (Choi, 2020)]. This excitotoxic cascade is one of the drivers of neuronal loss and glial activation in ischemic stroke and neurodegenerative diseases. Thus, understanding how the loss of IGF-1 alters the glutamate response of neurons, astrocytes, and endothelial cells within the neurovascular unit provides much-needed information on potential cellular mechanisms by which IGF-1 deficiency in advanced age influences health and function of the brain.

Recent evidence from our laboratory indicated that IGFR inhibition reduced the ability of astrocytes to buffer extracellular glutamate by decreasing glutamate transporter availability (Prabhu et al., 2019). Therefore, in this study, we hypothesized that maintenance of IGF-1 signaling in astrocytes is necessary for neuroprotection from glutamate excitotoxicity. We also hypothesized that maintenance of IGF-1 signaling in neurons was essential for limiting overexcitation, as other studies have shown exogenous IGF-1 can protect against excitotoxicity and oxidative stress (Wang et al., 2014; Li et al., 2017; Chen et al., 2019). Our results indicate that while exogenous IGF-1 can protect neurons from glutamate, acute and prolonged loss of IGFR signaling does not exacerbate neuronal death in cultures of pure neurons. Interestingly, inhibition of IGF-1R not only impairs the neuroprotective capabilities of astrocytes, it predisposes astrocytes to glutamate toxicity as well. It is unclear what the mechanism for this enhanced sensitivity may be. Perhaps the lack of glutamate transporter availability with IGF-1 signaling deficiency resulted in increased GluNR activation in the astrocytes as well, since astrocytes are known to express a variety of ionotropic and metabotropic GluNRs (Serrano et al., 2008; Lalo et al., 2011; Bradley and Challiss, 2012; Ceprian and Fulton, 2019). If this were the case, the astrocytes could be experiencing intracellular calcium imbalances and oxidative stress, which have both been shown to increase astrocyte toxicity in response to other stressors.

As mentioned, reductions in IGF-1 in the brain increase ROS levels and mitochondrial dysfunction. We observed significant increases in ROS production in both astrocytes and endothelial cells when IGFR was inhibited at the time of glutamate stimulation. We chose to focus our experimental design on these two cell types and this acute treatment because it did not lead to any changes in observed toxicity. Oxidative stress is expected in neurons that are dying from excitotoxicity, but neither astrocytes nor endothelial cells were sensitive to glutamate when IGFR was acutely inhibited. The increased ROS levels in both cell types indicates that the cells were indeed undergoing a stress response with the combined treatment. While it could be inferred that the ability of astrocytes to return ROS levels back to baseline by 24 h may contribute to their increased resistance to excitotoxic stress, there is a disconnect in the endothelial cells that likely renders this to be more complicated. Perhaps an increase in endothelial cell toxicity would have been observed at later time points. Further studies on the underlying mechanism(s) and consequence(s) of these observations are needed.

Combined triple cultures of neurons, astrocytes, and endothelial cells highlighted a few distinct responses to glutamate and IGFR inhibition than the individual cultures of each cell type. First, the same concentration of glutamate that led to significant increases in neurotoxicity within pure neuron cultures failed to induce toxicity within the triple culture. This was an expected outcome, as astrocytes are known to buffer glutamate and co-cultures of neurons and astrocytes have been shown to reduce the extent of neurotoxicity (Choi et al., 1987; Rosenberg et al., 1992). Second, and more interestingly, prolonged IGFR inhibition in astrocytes prior to glutamate stimulation did not increase the toxicity of astrocytes nor decrease the total cell number of astrocytes within the triple cultures. These effects were pronounced in pure astrocyte cultures, suggesting that co-culturing astrocytes with neurons and endothelial cells afforded them protection against the stressors of IGFR deficiency/glutamate stimulation. Additional studies examining ROS levels, inflammatory mediators, and growth factor production and release with triple cultures exposed to these same stressors may be warranted.

In our recent study examining glutamate uptake in astrocytes, we utilized similar concentrations of the pharmacological inhibitor PPP and did not observe a significant reduction in total cell number. Thus, we were surprised to see a reduction in astrocyte count here. However, in that study, cells were treated when at or close to confluence, which was evident in DAPI-stained microscopy images accompanying those data (Prabhu et al., 2019). In the current study, we aimed to treat at 75% confluence in order to avoid stress of over-growth, however, microscopic live/dead staining analysis showed that even the vehicle-treated controls were not at this level of confluence even 24 h after treatment. Thus, the reduction in cell number observed in pure cultures of astrocytes and endothelial cells was likely due to changes in cell division and growth with IGF-1 deficiency. Nevertheless, it is interesting that the reduction in astrocyte number with prolonged IGFR inhibition was restricted to the pure cultures, and not the triple cultures.

The complication of cell proliferation also limited our ability to utilize genetic approaches in our *in vitro* studies. IGF-1 is a primary growth factor with autocrine and paracrine functions, and genetic reductions that alter cell proliferation make it difficult to control studies of neuroendocrine hormones. We originally aimed to include siRNA and/or cell-specific knock-outs using our inducible cell-specific IGFR knock-out lines. However, due to obvious insufficient cell proliferation caused by siRNA transfection/transduction (both approaches were attempted) as well as Cre-recombinase induced knock-outs, we concluded that the significant reductions in cell number would confound results. Astrocyte count following knock-out with siRNA or with 4-OH-tamoxifen-induced recombination was less than 50% of that observed in the control-treated wells (siRNA vector transfected or vehicle-treated). Hence, pharmacological interventions were utilized to address our central question of how the loss of IGF-1 impacts the major cells of the neurogliovascular unit. While exogenous IGF-1 was added in a few studies here, the growth media of the cells contains insulin and IGF-1 at concentrations known to promote growth and development, thus pharmacological inhibitors were selected to more specifically reduce IGFR signaling. Alternative studies could alter the density of seeded cells to account for the loss in proliferation with knock-out, however, compensatory changes in other growth receptors (GH, InsR, etc.) and IGF-1 binding proteins will also need to be examined as they are known to occur with IGF-1 manipulations *in vivo* (Ashpole et al., 2015b).

Our comparative study is limited by not including additional cells found within the brain and blood-brain barrier. For example, pericytes not only serve as a protective layer of the blood brain barrier, they regulate blood capillary diameter within the brain by dilating and relaxing in response to glutamate (Kisler et al., 2017, 2020; Brown et al., 2019). Because of this, pericytes are important during ischemic reperfusion as they can assist with restoring flow of oxygen and glucose to the ischemic tissue (Yang et al., 2017; Khennouf et al., 2018; Sun J. et al., 2020). Little is known about the influence of IGF-1 on pericytes within the brain, and additional studies on how IGF-1 may influence pericytic glutamate response are needed.

Additionally, microglia are known to respond to excitotoxic insult, ischemia, and reperfusion. In fact, microglia have been implicated in the exacerbation of damage following glutamate excitotoxicity and ischemic stroke, due to increased production of ROS and pro-inflammatory mediators (Ma et al., 2017; Qin et al., 2019; Lian et al., 2020). At the same time, microglia also play an important protective role following tissue stroke damage, so the duality of their contribution to physiology/pathophysiology cannot be over-simplified to just a negative contribution to damage (Ma et al., 2017; Qin et al., 2019; Marino Lee et al., 2021). The age-related loss of circulating IGF-1 is associated with increased microglial activation, neuroinflammation, and susceptibility to a compromised blood-brain barrier. The relationship between IGF-1 and microglial activation is not limited to temporal correlations, as there are numerous studies that highlight IGF-1 directly regulates the structure and function of microglia. Exogenous IGF-1 alters phenotypic activation states and reduces microglial-associated production and release of tumor necrosis factor-alpha (TNF-α), interleukin-1 beta (IL-1β), inducible nitric oxide synthase (iNOS), and ROS (Park et al., 2011; Grinberg et al., 2013). Administration of IGF-1 following ischemic and/or hemorrhagic stroke attenuates microglial activation, inflammation, and ROS (Serhan et al., 2020; Sun Z. et al., 2020). Considering this, we expect that reductions in IGF-1 signaling within our cell culture system would also promote pro-inflammatory and oxidative stress phenotypes, which would further neuronal death. However, one study highlights that microglial presence in tri-cultures are neuroprotective and reduces neurons loss and astrocyte hypertrophy even though the presence of microglia increased inflammatory cytokine production compared to cell culture without microglia (Goshi et al., 2020). Future studies of ischemic and excitotoxic stressors should take microglial contributions into account while controlling for the natural migratory phenotype microglia display following activation, as results may be confounded by increased basal levels of neuroinflammatory stress (which often show an activated phenotype in culture).

Transitioning to an *in vivo* system would circumvent concerns regarding the lack of including all cell types present in the cerebral tissue within our co-culture models; however, the intricacies of timed, cell-specific manipulations make *in vivo* models difficult, particularly when trying to assess effects without developmental compensation interference. Inducible transgenic animals for each cell type are needed to rigorously assess the effects of the loss of IGF-1 in adulthood on each component of the neuro-glio-vascular unit. We previously established an inducible astrocyte-specific transgenic mouse model, and identified that glutamate handling machinery was reduced in the brains of these mice, which served as a rationale for studying glutamate excitotoxicity here (Prabhu et al., 2019). A recent study highlighted changes in neurovascular coupling when IGFR signaling was targeted in adult microvascular endothelial cells (Tarantini et al., 2021b). Thus, inducible models of astrocytic and endothelial cell IGFR knock-outs are available. Our singular and co-culture *in vitro* studies allow us to begin to delineate and compare the responses of specific cells without the confounds of other variables like paracrine compensation or pharmacological drug delivery or metabolism in one cell type over another. Further side-by-side comparisons of excitotoxic or ischemic damage in mice with inducible IGFR knock-out in neurons, astrocytes, endothelial cells, pericytes, and microglia would confirm the role for IGF-1 regulation of each cell, while also revealing other potential changes in physiology and pathophysiological responses in the intact brain.

Together, our study highlights that cell types within the neurovascular unit differentially respond to IGF-1 signaling. Despite differences in baseline susceptibility to glutamate, both neurons and astrocytes are both protected from excitotoxicity by IGF-1. Growth and division of endothelial cells and astrocytes are both influenced by IGF-1, however, endothelial cells remain resistant to glutamate toxicity even when IGF-1 signaling is reduced. The resistance of these supporting cells does not mean there are no consequences of reduced IGF-1 signaling in the time of glutamate stress, as both astrocytes and endothelial cells show signs of oxidative stress in the hours following exposure. The combination of neuron, astrocyte, and endothelial cells in culture afforded astrocytic protection from IGF-1 deficiency/glutamate stress but also highlighted a failure in the ability of astrocytes to protect the nearby neurons. Thus, the age-related loss of IGF-1 likely impairs the function and vitality of the entire neurovascular unit by differentially exerting stressors on neurons, endothelial cells, and astrocytes.

## Data Availability Statement

The raw data supporting the conclusions of this article will be made available by the authors, without undue reservation.

## Ethics Statement

The animal study was reviewed and approved by Institutional Animal Use and Care Committee of the University of Mississippi.

## Author Contributions

CH and NA were responsible for conceptualization, investigation, project administration, and formal statistical analyses. CH, JM, and NA developed and designed methodology. BA, AV, and NA performed blinded image analyses and curated data. CH prepared visualizations. CH, BA, and NA prepared the original draft. All authors reviewed and edited the manuscript.

## Conflict of Interest

The authors declare that the research was conducted in the absence of any commercial or financial relationships that could be construed as a potential conflict of interest.

## Publisher’s Note

All claims expressed in this article are solely those of the authors and do not necessarily represent those of their affiliated organizations, or those of the publisher, the editors and the reviewers. Any product that may be evaluated in this article, or claim that may be made by its manufacturer, is not guaranteed or endorsed by the publisher.

## Supplementary Material

Supplementary Figure 1 Additional assessments of total cell number. **(A)** Average number of neurons per field 24 h after treatment. One-way ANOVA revealed no difference between means. **(B–D)** Astrocytes were pre-treated with 5 μM PPP for 24 h prior to combining with endothelial cells and neurons for a triple culture system. Average number of cells per field in the neurons **(B)**, astrocytes **(C)**, and endothelial cells **(D)** cultures after stimulation with 100 μM glutamate for 1 h (*n* = 5–8 wells/group). One-way ANOVA revealed no difference between means, and the *p* value of the ANOVA is listed for each. All data are presented as mean ± SEM. **(E)** Average astrocyte viability 24 h after 100 μM glutamate stimulation. Prior to stimulation, the cells were pre-treated with vehicle, 5 μM NVP-ADW742, or 100 nM IGF-1 for 24 h (*n* = 6–8 wells/group). **(F)** Average astrocyte cell count following treatment with vehicle, 5 μM NVP-ADW742, or 100 nM IGF-1 (*n* = 6–8 wells/group).

Supplementary Figure 2 Astrocyte ROS and mitochondrial stress. **(A)** Average ROS levels 5 h after pure cultures of astrocytes treated with 0.5 μM NVP-ADW742 and 100 μM glutamate. Data were not normally distributed, so a Tukey’s *post hoc* comparison was utilized following One-way ANOVA; ^∗^ indicates a significant difference compared to vehicle (*n* = 10 wells/group). **(B)** Average oxygen consumption rate (*y* axis) and extracellular acidification rate (*x* axis) in astrocytes treated with vehicle control, 100 μM glutamate, or 100 μM glutamate + 0.5 μM PPP for one hor. Measurements occurred 5 h after treatment. Maximal respiration is grouped in the top right 3 points, and basal respiration is grouped in the bottom left 3 points. A one-way ANOVA was used for each comparison of maximal OCR, maximal ECAR, basal OCR, and basal ECAR. *Post hoc* Dunnett’s test vs vehicle control was used when relevant. ^∗^ indicates significant difference in OCR, and ^#^ indicates significant difference in ECAR. All data are presented as mean ± SEM, *n* = 7–8 wells/group.
