# Supplementary figures and images for: Insulin-Like Growth Factor-1 Differentially Modulates Glutamate-Induced Toxicity and Stress in Cells of the Neurogliovascular Unit
Source: Front Aging Neurosci. 2021 Nov 23;13:751304. doi: 10.3389/fnagi.2021.751304 (PMC8650493; doi:10.3389/fnagi.2021.751304)

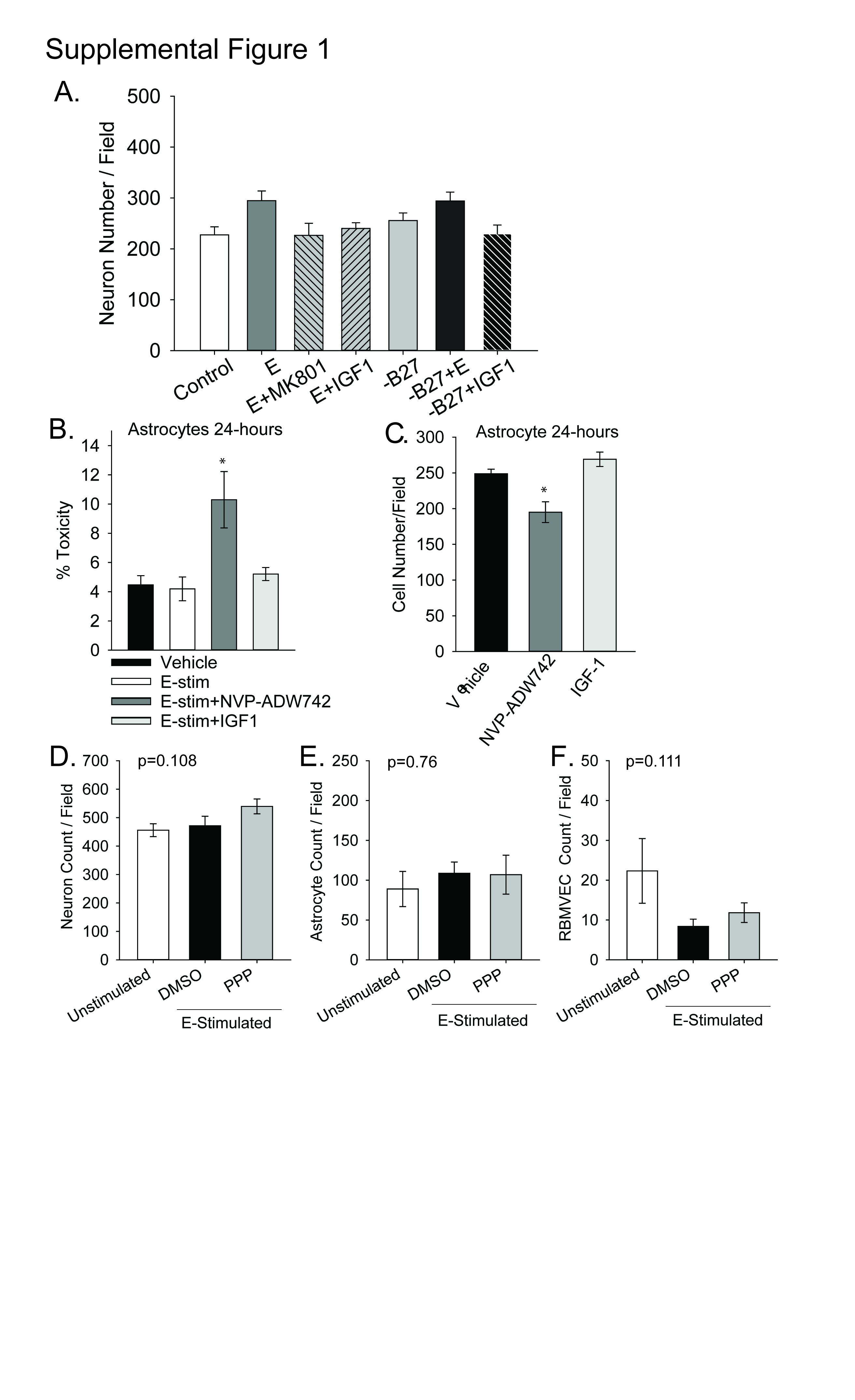

Supplement: Supplementary Figure 1 — Additional assessments of total cell number. (A) Average number of neurons per field 24 h after treatment. One-way ANOVA revealed no difference between means. (B–D) Astrocytes were pre-treated with 5 μM PPP for 24 h prior to combining with endothelial cells and neurons for a triple culture system. Average number of cells per field in the neurons (B), astrocytes (C), and endothelial cells (D) cultures after stimulation with 100 μM glutamate for 1 h (n = 5–8 wells/group). One-way ANOVA revealed no difference between means, and the p value of the ANOVA is listed for each. All data are presented as mean ± SEM. (E) Average astrocyte viability 24 h after 100 μM glutamate stimulation. Prior to stimulation, the cells were pre-treated with vehicle, 5 μM NVP-ADW742, or 100 nM IGF-1 for 24 h (n = 6–8 wells/group). (F) Average astrocyte cell count following treatment with vehicle, 5 μM NVP-ADW742, or 100 nM IGF-1 (n = 6–8 wells/group). [file Image_1.tif]

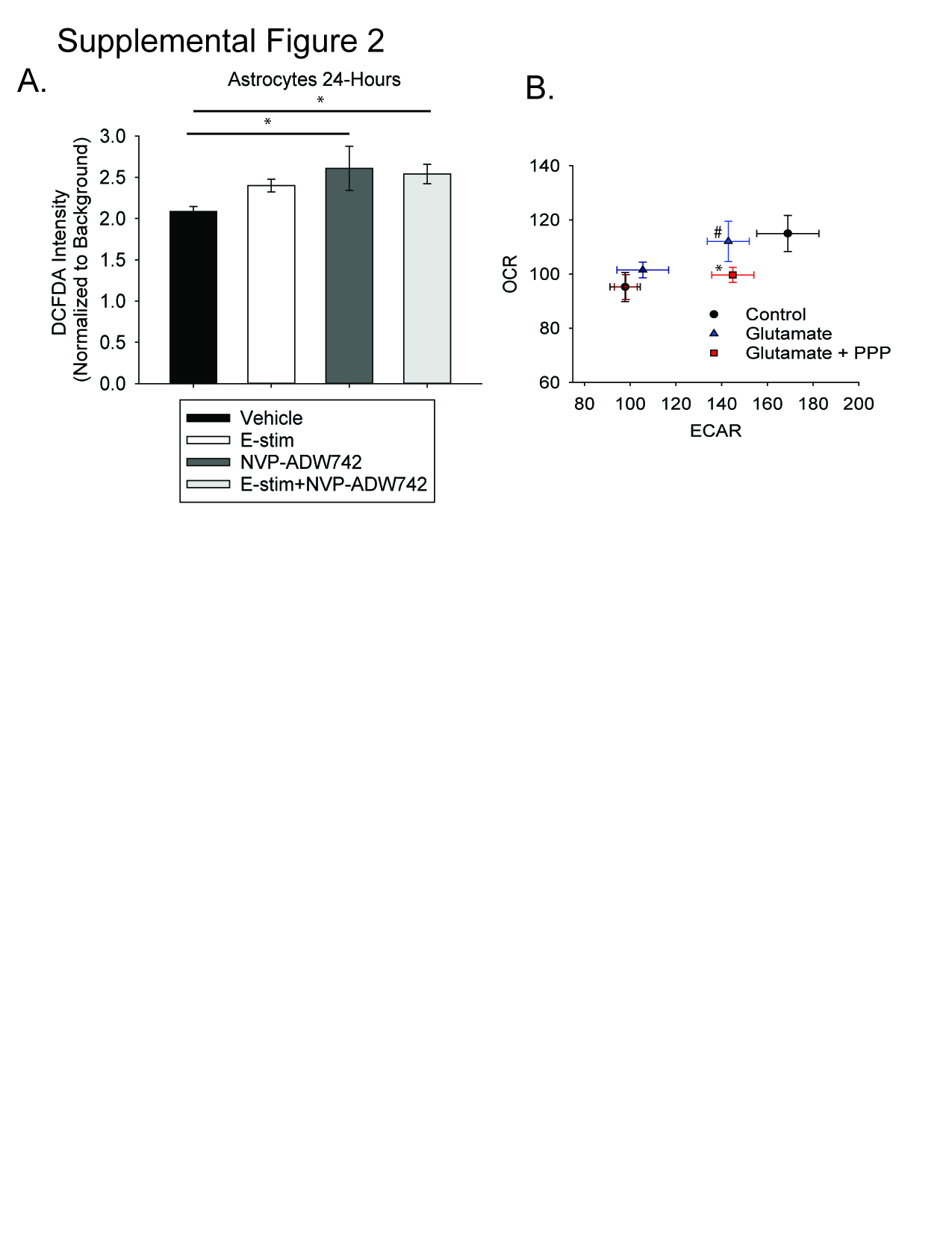

Supplement: Supplementary Figure 2 — Astrocyte ROS and mitochondrial stress. (A) Average ROS levels 5 h after pure cultures of astrocytes treated with 0.5 μM NVP-ADW742 and 100 μM glutamate. Data were not normally distributed, so a Tukey’s post hoc comparison was utilized following One-way ANOVA; ∗ indicates a significant difference compared to vehicle (n = 10 wells/group). (B) Average oxygen consumption rate (y axis) and extracellular acidification rate (x axis) in astrocytes treated with vehicle control, 100 μM glutamate, or 100 μM glutamate + 0.5 μM PPP for one hor. Measurements occurred 5 h after treatment. Maximal respiration is grouped in the top right 3 points, and basal respiration is grouped in the bottom left 3 points. A one-way ANOVA was used for each comparison of maximal OCR, maximal ECAR, basal OCR, and basal ECAR. Post hoc Dunnett’s test vs vehicle control was used when relevant. ∗ indicates significant difference in OCR, and # indicates significant difference in ECAR. All data are presented as mean ± SEM, n = 7–8 wells/group. [file Image_2.tif]
